# Relationship between oral declaration on adherence to ivermectin treatment and parasitological indicators of onchocerciasis in an area of persistent transmission despite a decade of mass drug administration in Cameroon

**DOI:** 10.1186/s13071-015-1283-6

**Published:** 2015-12-30

**Authors:** Samuel Wanji, Jonas A. Kengne-Ouafo, Mathias E. Esum, Patrick W. N. Chounna, Bridget F. Adzemye, Joan E. E. Eyong, Isaac Jato, Fabrice R. Datchoua-Poutcheu, Raphael A. Abong, Peter Enyong, David W. Taylor

**Affiliations:** Parasite and Vectors Research Unit, Department of Microbiology and Parasitology, University of Buea, P.O.Box 63, Buea, Cameroon; Research Foundation for Tropical Diseases and Environment, P.O.Box 474, Buea, Cameroon; Tropical Medicine Research station, P.O. Box 55, Kumba, Cameroon; Department of Biological Sciences, Faculty of Science, University of Bamenda, P.O. Box 39, Bambili, North West Region Cameroon; Division of Infection and Pathway Medicine, School for Biomedical Studies, University of Edinburgh, 49 Little France Crescent, Edinburgh, EH16 4SB UK

**Keywords:** Oral declaration, Adherence, Ivermectin, Parasitological indicators, Transmission, MDA, Onchocerciasis

## Abstract

**Background:**

Onchocerciasis control for years has been based on mass drug administration (MDA) with ivermectin (IVM). Adherence to IVM repeated treatment has recently been shown to be a confounding factor for onchocerciasis elimination precisely in rain forest areas where transmission continues and *Loa loa* co-exists with *Onchocerca volvulus*. In this study, participants’ oral declarations were used as proxy to determine the relationship between adherence to IVM treatment and parasitological indicators of onchocerciasis in the rain forest area of Cameroon with more than a decade of MDA.

**Methods:**

Participants were recruited based on their IVM intake profile with the aid of a semi-structured questionnaire. Parasitological examinations (skin sniping and nodule palpation) were done on eligible candidates. Parasitological indicators were calculated and correlated to IVM intake profile.

**Results:**

Of 2,364 people examined, 15.5 % had never taken IVM. The majority (40.4 %) had taken the drug 1–3 times while only 18 % had taken ≥ 7 times. Mf and nodule prevalence rates were still high at 47 %, 95 % CI [44.9–49.0 %] and 36.4 %, 95 % CI [34.4–38.3 %] respectively. There was a treatment-dependent reduction in microfilaria prevalence (r_s_ =−0.986, P = 0.01) and intensity (r_s_ =−0.96, P = 0.01). The highest mf prevalence (59.7 %) was found in the zero treatment group and the lowest (33.9 %) in the ≥ 7 times treatment group (OR = 2.8; 95 % CI [2.09–3.74]; P < 0.001). Adults with ≥ 7 times IVM intake were 2.99 times more likely to have individuals with no microfilaria compared to the zero treatment group (OR = 2.99; 95 % CI [2.19–4.08], P < 0.0001). There was no clear correlation between treatment and nodule prevalence and intensity.

**Conclusion:**

Adherence to ivermectin treatment is not adequate in this rain forest area where *L. loa* co-exists with *O. volvulus*. The prevalence and intensity of onchocerciasis remained high in individuals with zero IVM intake after more than a decade of MDA. Our findings show that using parasitological indicators, reduction in prevalence is IVM intake-dependent and that participants’ oral declaration of treatment adherence could be relied upon for impact studies. The findings are discussed in the context of challenges for the elimination of onchocerciasis in this rain forest area.

## Background

Onchocerciasis is a debilitating vector-borne disease caused by the parasite *Onchocerca volvulus*. It is transmitted by black flies of genius *Simulium* [1, 2]. The disease is a public health and socio-economic threat in many African countries [3–5]. It has been estimated that 36 million people are infected [6] and 86 million people live in high risk areas in the African programme for Onchocerciasis control (APOC) countries [7]. Onchocerciasis is the second-leading infectious cause of blindness worldwide, being responsible for about 500,000 cases of blindness [8, 9] Using the Rapid Epidemiological Mapping of Onchocerciasis (REMO), different foci have been mapped out in Cameroon [10] including the rain forest of South West Cameroon which is co-endemic with loiasis [11].

Since the creation of APOC in 1995, the control of onchocerciasis has been based on annual mass drug distribution (MDA) in most endemic countries [12]. This was done by establishing the Community-Directed Treatment with ivermectin (CDTI). With CDTI, drug distribution is carried out by selected community members who share the drug annually and make records for eventual evaluation of the programme if treatment registers are well kept. The main objective of APOC was to reduce the prevalence and transmission of onchocerciasis to a point where the disease will no longer be a public health problem in countries not previously covered by the Onchocerciasis Control Programme (OCP). A single annual dose of ivermectin can clear microfilariae from the skin and consequently reduce morbidity associated with the infection. Although ivermectin is an extremely effective microfilaricide, many years of administration are required to kill adult worms. Hence onchocerciasis elimination depends to a great extent to adherence to repeated annual treatment with ivermectin [13, 14]. Adherence or compliance refers to the extent to which a patient acts in accordance with the prescribed interval and dosing regimen [15]. In some contexts, the two terms are used interchangeably however, the main difference is that adherence requires the patient’s agreement to the recommendations [16]. Since with CDTI, participants are not forced to take the drug but instead are sensitised and educated on its benefits, the term adherence seems more relevant.

The feasibility and purported elimination of onchocerciasis has been demonstrated in some savannah regions of Africa after 15-17 years of treatment [17–20]. However, the situation is different in rain forest areas as demonstrated by Wanji and colleagues [21] where transmission of the parasite has continued even after a decade of MDA. This persistent transmission could be due to low adherence to ivermectin treatment because of fear of severe adverse effects [14, 22–24]. It could also be due to ecological factors that strongly favour transmission of *O. volvulus* [21].

In a multi-site study evaluating five CDTI projects in Nigeria and Cameroon, where at least eight annual IVM distributions had taken place, it was demonstrated that over one-quarter of age-eligible people in study communities were low compliers [25] and thus serve as a reservoir for continued transmission of onchocerciasis [14, 26]. Reports of the impact of adherence to ivermectin treatment on parasitological indicators are scarce or absent from rain forest areas. This may be explained by the absence or poor quality of treatment registers. Records kept by community drug distributors (CDDs) are often incomplete and therefore considered unreliable to assess adherence [27].

In this study, participants’ oral declarations were used as proxy to determine the relationship between adherence to ivermectin treatment and parasitological indicators of onchocerciasis in the rain forest area of Cameroon with more than a decade of CDTI. This approach has been used in a small number of other studies [13, 14, 27].

## Methods

### Study site

The study was carried out in communities in the Konye, Mamfe, Eyumojock and Kumba health districts, which are located in three different hydrographical basins situated in the rain forest zone of the South West, Cameroon and previously described by Wanji and colleagues [21]. The climate of the study sites is of the equatorial type. The temperatures are high, ranging from 25 to 32 °C. The vegetation is secondary forest resulting from the degradation of the dense humid forest for agricultural activities. The tributaries of the Rivers Mungo and Meme (Konye and Kumba) and the tributaries of River Manyu (Mamfe and Eyumojock) serve as breeding sites for *Simulium* spp. in these areas.

### Study population and design

This cross-sectional study was designed to assess the relationship between ivermectin treatment adherence and parasitological indices of onchocerciasis [21]. Community-Directed Treatment with Ivermectin (CDTI) had been going on in the study area for 10 to 12 years with geographical coverage varying between 95–100 % and therapeutic coverage generally above 65 % [21]. Some cases of severe adverse reactions had been reported in this area at the onset of ivermectin treatment [28]. Participants enrolled in this study were from communities found around the three drainage basins. They were of both sexes and aged 5-94 years. They had not taken any filaricide medication for one year prior to sample collection but had been resident in the area for at least five years. Participants were recruited with the aid of a structured questionnaire and all had given their consent or assent (children) to take part in the study.

In addition to demographic data, questions were asked as to whether individuals had ever taken ivermectin and the number of times and the last time (s)he had taken the drug. Comparison of the records of the oral declarations with the records of the CDDs treatment registers was not done because the latter were incomplete and therefore considered unreliable. Eligible participants were categorised by the number of times they had taken the drug (zero; 1 – 3; 4 – 6; and, ≥ 7 times). All were subjected to a parasitological examination and the results obtained correlated with their treatment profiles. Only participants who had not taken any filaricide medication for the past one year were enrolled in the study to avoid the situation where a reduction in mf load could be due to recently administered drug. Recruitment of this cohort was helped by beginning the study before the large scale annual distribution of ivermectin. Information on the CDTI indicators (geographical and treatment coverages) were obtained from the National Onchocerciasis Control Programme through their regional branches in the south west. Parasitological surveys were carried out in April and July of 2011 and 2012. Mf and nodule prevalence and Mf and nodule intensity were recorded against ivermectin treatment profiles.

### Ethical considerations

Study was approved by the Cameroon National Ethics Committee and the Ministry of Public Health. Individuals recruited as volunteers were informed of the study protocol, and the importance of the study for the improvement of control measures. Participants were also told about potential risks to which they could be exposed and the benefits they could receive during and after the study. Only those who indicated their consent by signing a consent or assent form were recruited for the study. Recruitment was done based on the approach described previously by Kengne-Ouafo et al. [29] after a rapid ethical assessment undertaken in the North-West Cameroon to deteremine the level of individual and community knowledge of ethical issues related to biomedical research. All participants’ information collected during the course of the research were kept on a password protected database and were strictly confidential.

### Parasitological examinations

Parasitological examinations were carried out as previously described by Wanji et al. [21]. Nodule palpation and skin snipping were carried out to determine the presence of the parasite. This was done on partially undressed patients following Rapid Epidemiological Assessment (REA) guidelines [6, 7, 10, 30]. Attention was paid to bony prominences of the iliac crest, torso, knees, arms, head and the upper trochanter of the femur. The number of nodules found and their position were recorded.

From each patient, 2 skin biopsies, one from each upper iliac crest were taken using a sharp sterile sclera punch (CT 016 Everhards 2218-15 C, Germany). The biopsies were immediately placed into physiological saline in separate wells of a 96-well culture plate. The corresponding well numbers were reflected on the participant’s form. The plates were sealed with parafilm to prevent any spill over or evaporation. Observation and microfilaria count was done using an inverted microscope (Motic AE21) at magnification of 10× 24 h later [21, 31, 32]. Microfilaria count was expressed as mf/skin snip (ss).

### Data analysis

Data collected were entered in a template created in EPI INFO 6 (version 6) and were analysed using SPSS 20 (Software SPSS INC, Chicago, IL, USA) and Microsoft Excel 2013. Individuals were selected and categorised by: (i) sex (male and female); (ii) age groups [children (<20 year-old) and adults (≥20 year-old)]; (iii) ivermectin or IVM intake defined as the number of times a participant had taken ivermectin (zero, i.e. have never taken ivermectin; 1-3 treatments; 4-6 treatments; and ≥7 treatments); (iv) microfilarial load (zero, i.e. no mf load; 1–50 mf/ss; 51–100 mf/ss; and ≥ 100 mf/ss); (v) nodule number (0, 1, 2, ≥3). Adults were defined as individuals 20 year-old and above because this age group is used in the computation of the community microfilarial load (CMFL). Those of the < 20 year-old age group were identified as children, and could not be split further because of the limited number of persons within the study cohort. CMFL was expressed as the geometric mean. The intensity of infection in other groups or categories was referred to as Williams mean microfilaria density (WMMfD) as reported previously [21]. The calculation was done using the log (x +1) transformation, where x is the individual microfilaria load [10]. Contingency tables were used to express the association between variables. The Chi-square test were also used to compare the prevalence rates of infection between categories. Student *t*-test and ANOVA were used to compare means between sexes, age groups and IVM intake groups. Spearman’s rank correlation was used to show the association between IVM intake and Mf prevalence; and intensity. Binary logistic regression analysis was used to check whether IVM intake was a significant factor in onchocerciasis prevalence and intensity reduction. All the statistical tests were performed at a 5 % significance level.

## Results

### Study population characteristics

A total of 2,364 (1,269 males and 1,095 females) participants were enrolled in the study. The mean age of the study participants was 35.9 years ranging from 5 to 100 years. The study participants were composed of 477 Children [<20 year-old] and 1,887 adults [20 year-old and above).

### Ivermectin intake profile of the study population

A total of 1,997 participants had taken ivermectin at least once out of the 10–12 rounds of distribution making an overall adherence rate of 84.47 %. Of the 2,364 individuals examined, 367 (15.53 %) had never taken ivermectin, 956 (40.41 %) had taken between 1-3 times, 615 (26.02 %) had taken between 4-6 times and 426 (18.0 %) had taken ≥ 7 times. There was a significant difference in the frequency distribution of participants between IVM intake groups (Table 1, P = 0.031). The majority of children (66.2 %) had taken ivermectin 1-3 times compared to 33.9 % for adults. However just few children/adolescents (1.5 %) had received the drugs more than 7 times compared to 22.2 % for adults.

**Table 1:** Ivermectin intake based on oral declarations in the study population by age and sex

		IVM INTAKE					
	Gender	0	[1–3]	[4–6]	>7	Total	
Children	Male	42 (16.2)	179 (69.1)	35 (13.5)	3 (1.2)	259	
	Female	40 (18.3)	137 (62.8)	37 (17.0)	4 (1.8)	218	
	Total	82 (17.2)	316 (66.2)	72 (15.1)	7 (1.5)	477	*P* = 0.508
Adults	Male	169 (16.7)	347 (34.4)	265 (26.2)	229 (22.7)	1010	
	Female	116 (13.2)	293 (33.4)	278 (31.7)	190 (21.7)	877	
	Total	285 (15.1)	640 (33.9)	543 (28.8)	419 (22.2)	1887	*P* = 0.029
Total	Male	211 (16.6)	526 (41.4)	300 (23.7)	232 (18.3)	1269	
	Female	156 (14.2)	430 (39.3)	315 (28.8)	194 (17.7)	1095	
	Total	367 (15.5)	956 (40.4)	615 (26.0)	426 (18.0)	2364	*P* = 0.031

*IVM* Ivermectin

Sex did not appear to be a critical factor influencing IVM intake: of 1,269 men who took part in the study, 211 (16.6 %) had never taken ivermectin and 156/1,095 (14.2 %) females had never taken the drug (P = 0.1245). The same trend was seen in all the treatment groups (Table 1, P > 0.05).

### *O. volvulus* infection in the study population

#### Microfilaria and nodule prevalence

Of 2,364 persons that took part in the study, 1,110 (47.0 %, 95 % CI [44.9–49.0 %]) were mf positive (Table 2). Although more males were infected 48.6 %, 95 % CI [45.8–51.3 %] than females 45.0 %, 95 % CI [42.0–47.9 %], the difference was not significant (P = 0.08). Similar observations were made for adults (P = 0.173) and children (P = 0.246). Mf prevalence was relatively higher in children than adults (49.7 %, 95 % CI [45.2–54.1 %] *vs* 46.3 %, 95 % CI [44.0–48.5 %]; P = 0.181; Table 2).

**Table 2:** Observed parasitological indices of onchocerciasis in the study population by age and sex

	Gender	Number examined	Microfilaria prevalence	Nodule prevalence	WMMfD (Mf/ss)
Children	Male	259	135 (52.1)	74 (28.6)	5.62
	Female	218	102 (46.8)	45 (20.6)	4.75
	Total	477	237 (49.7)	119 (24.9)	5.22
Adults	Male	1010	482 (47.7	436 (43.2)	5.52
	Female	877	391 (44.6)	305 (34.8)	3.97
	Total	1887	873 (46.3)	741 (39.3)	4.72
Total	Male	1269	617 (48.6	510 (40.2)	5.54
	Female	1095	493 (45.0)	350 (32.0)	4.11
	Total	2364	1110 (47.0	860 (36.4)	4.82

*WMMfD* Williams mean microfilaria density

The overall nodule prevalence was 36.4 %, 95 % CI [34.4–38.3 %]. Nodules were significantly more prevalent in males than in females (40.2 %, 95 % CI [37.5–42.9 %] *vs* 32.0 %, 95 % CI [29.2–34.7 %]; P < 0.001; Table 2). As observed in the case of mf prevalence, nodule prevalence was higher in males than in females, both in children and adults (P = 0.046 and P < 0.001, respectively). Nodules were rather more prevalent in adults than in children (P < 0.001; Table 2).

#### Microfilaria and nodule intensity

The overall Williams mean mf density (WMMfD) was 4.8. The infection was more intense in males than in females, both in children and adults (Table 2, P < 0.001). The WMMfD was higher in children than in adults but the difference was not significant (Table 2; P = 0.454). Of the examined population, 1,010 (42.7 %) had mf load between 1–50 mf/ss, 51 (2.2 %) between 51–100 mf/ss and 49 (2.1 %) greater than 100 mf/ss. These proportions were slightly higher in males than in females (Table 3). There was a significant difference in the proportions of individuals between the various mf load groups (P = 0.017) but not within groups (P > 0.05). This profile was seen both in children and adults (Table 3). The majority (53.0 %) of the study population had no mf in their biopsies.

**Table 3:** Proportions of individuals in defined mf load groups by age and sex

		Mf load group					
	Gender	0mf	1-50mf	51-100mf	>100mf	Total	
Children	Male	124 (47.9)	118 (45.6)	9 (3.5)	8 (3.1)	259	
	Female	116 (53.2)	94 (43.1)	4 (1.8)	4 (1.8)	218	
	Total	240 (50.3)	212 (44.4)	13 (2.7)	12 (2.5)	477	*P* = 0.434
Adults	Male	528 (52.3)	431 (42.7)	27 (2.7)	24 (2.4)	1010	
	Female	486 (55.4)	367 (41.8)	11 (1.3)	13 (1.5)	877	
	Total	1014 (53.7)	798 (42.3)	38 (2.0)	37 (2.0)	1887	*P* = 0.056
Total	Male	652 (51.4)	549 (43.3)	36 (2.8)	32 (2.5)	1269	
	Female	602 (55.0)	461 (42.1)	15 (1.4)	17 (1.6)	1095	
	Total	1254 (53.0)	1010 (42.7)	51 (2.2)	49 (2.1)	2364	*P* = 0.017

The mean number of nodule among positive individuals was 1.64 and ranged between 1–11. Only 5.6 % of the study population had more than 3 nodules compared to 63.6 %, 21.8 % and 8.9 % with 0, 1 and 2 nodule, respectively. The same trend was observed among males and females, both in children and adults (Table 4, P < 0.001).

**Table 4:** Frequency distribution of the study population in defined nodule load groups by age and sex

		Number of nodule					
	Gender	0	1	2	>3	Total	
Children	Male	185 (71.4)	54 (20.8)	12 (4.6)	8 (3.1)	259	
	Female	173 (79.4)	29 (13.3)	12 (5.5)	4 (1.8)	218	
	Total	358 (75.1)	83 (17.4)	24 (5.0)	12 (2.5)	477	*P* = 0.123
Adults	Male	574 (56.8)	244 (24.2)	119 (11.8)	73 (7.2)	1010	
	Female	572 (65.2)	189 (21.6)	68 (7.8)	48 (5.5)	877	
	Total	1146 (60.7)	433 (22.9)	187 (9.9)	121 (6.4)	1887	*P* = 0.001
Total	Male	759 (59.8)	298 (23.5)	131 (10.3)	81 (6.4)	1269	
	Female	745 (68.0)	218 (19.9)	80 (7.3)	52 (4.7)	1095	
	Total	1504 (63.6)	516 (21.8)	211 (8.9)	133 (5.6)	2364	*P* < 0.001

### Influence of Treatment on *O. volvulus* infections

#### Effect of ivermectin treatment on O. volvulus mf and nodule prevalence

**Fig. 1:**
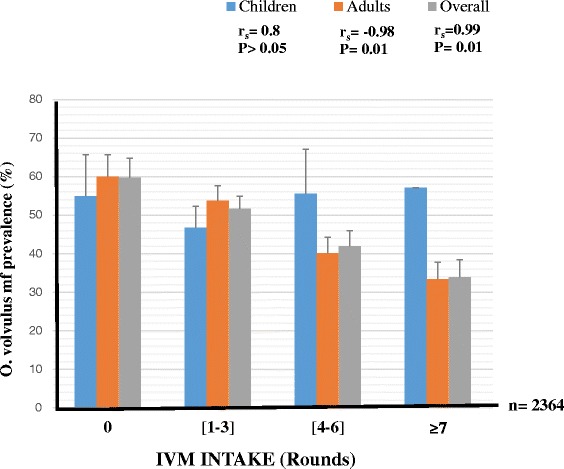
Effect of ivermectin treatment on *O. volvulus* mf prevalence in the study population. (Number of people examined per IVM intake groups written in the order children, adults (overall): [0 time] = 82, 285 (367); [1-3 times] = 316, 640 (956); [4-6 times] = 72, 543 (615); [≥7 times] = 7, 419 (426). Bars represent the 95 % margin of error. Significance level set at 5 %

Globally, there was a negative association between *O. volvulus* mf prevalence and the ivermectin intake (r_s_ = -0.99, P = 0.01). The highest mf prevalence (59.7 %) was found in the zero treatment group (i.e. people who had never taken ivermectin). This prevalence gradually declined with treatment to 33.9 % in the ≥ 7 times treatment group (OR = 2.8; 95 % IC [2.09–3.74]; P < 0.001). This trend showed that the more ivermectin is taken the lower the prevalence (Fig. 1). However, no such association was found in children (Fig. 1).

**Fig. 2:**
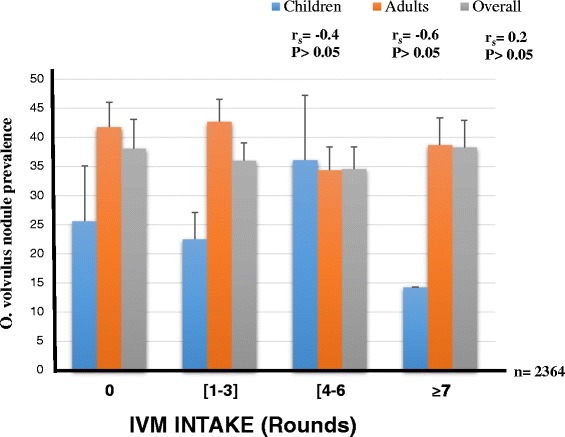
Effect of ivermectin treatment on *O. volvulus* nodule prevalence in the study population. (Number of people examined per IVM intake groups written in the order children, adults (overall): [0 time] = 82, 285 (367); [1-3 times] = 316, 640 (956); [4-6 times] = 72, 543 (615); [≥7 times] = 7, 419 (426). Bars represent the 95 % margin of error. Significance level set at 5 %

In contrast, no association was found between the overall nodule prevalence and IVM treatment. Nodule prevalence for individuals who had never received the drug was similar to that of those who had received it 7 times or more (38.1 % *vs* 38.3 %; Fig. 2) and there was no significant difference in overall nodule prevalence between the different IVM intake groups (P = 0.568). The same observation was made for children (P = 0.09). However, there were significant variations (with no trend) in nodule prevalence with respect to IVM intake for adults (P = 0.02, Fig. 2).

#### Effect of ivermectin treatment on O. volvulus mf and nodule intensity

**Fig. 3:**
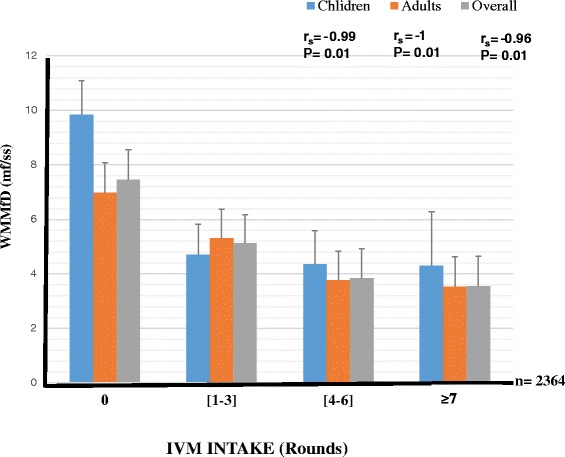
Effect of ivermectin treatment on *O. volvulus* mf intensity expressed as the Williams mean mf density. (Number of people examined per IVM intake groups written in the order children, adults (overall): [0 time] = 82, 285 (367); [1-3 times] = 316, 640 (956); [4-6 times] = 72, 543 (615); [≥7 times] = 7, 419 (426). Bars represent the standard error of the mean. Significance level set at 5 %

The intensity of infection (WMMfD) was observed to be high in the group of people who had never taken Ivermectin with an overall mean of 7.46. Children had the highest WMMfD (9.84) in this IVM intake group (Fig. 3). There was a significant decrease in WMMfD with ivermectin intake irrespective of age. The greater the number of times an individual had taken the drug, the lower his or her WMMfD (F = 18.99; P < 0.001). There was a negative correlation between IVM intake and intensity of infection (r_s_ = -0.96, P < 0.001, r_s_ = -1.00, P = 0.01, and r_s_ = -0.99, P = 0.01 for the overall study population, adults and children, respectively; Fig. 3).

**Fig. 4:**
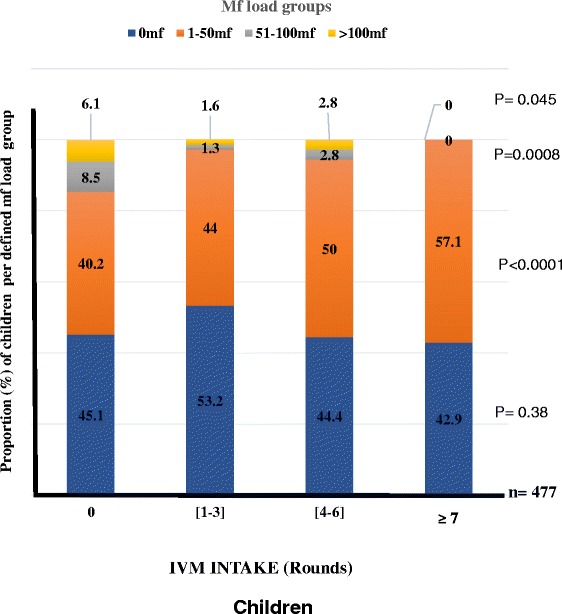
Changes in the proportion of children in defined mf load groups in relation to IVM intake profile. (Number of people examined per IVM intake group): [0 time] = 82; [1-3 times] = 316; [4-6 times] = 72 [≥7 times] = 7). P-value given for each defined mf load group and significance level set at 5 %

**Fig. 5:**
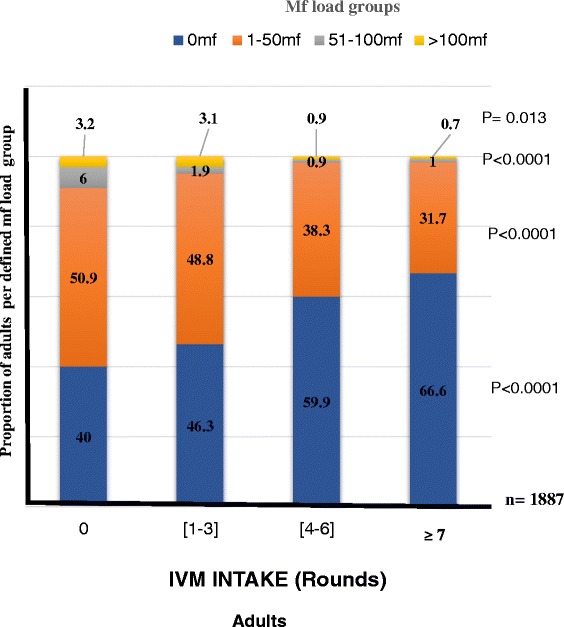
Changes in the proportion of adults in defined mf load groups in relation to IVM intake profile. (Number of people examined per IVM intake groups: [0 time] = 285; [1-3 times] = 640; [4-6 times] = 543; [≥7 times] = 419. P-value given for each defined mf load group and significance level set at 5 %

**Fig. 6:**
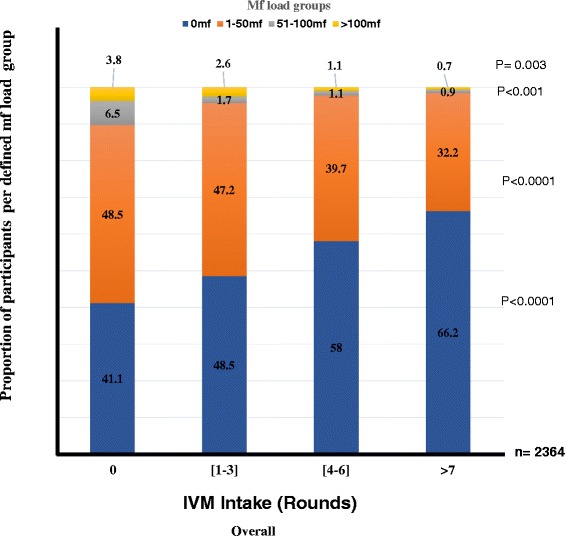
Changes in the proportions of study population in defined mf load groups in relation to IVM intake profile. (Number of people examined per IVM intake groups: [0 time] = 367; [1-3 times] = 956; [4-6 times] =615; [≥7 times] =426). P-value given for each defined mf load group and significance level set at 5 %

Like with mf prevalence, no association with trend was found between IVM intake and the proportion of individuals with different mf loads for children (Fig. 4). However, the proportion of people with no microfilariae in the skin was found to increase with IVM intake in adults and overall study population (Figs. 5 and 6). Of the 285 (15.1 %) adults who had never taken the drug, 40.0 % had no microfilaria. This proportion gradually increased with IVM intake to 66.6 % for those who had taken the drug 7 times and above (Fig. 5, P < 0.0001). Logistic regression revealed that adults who had had IVM 7 times or more were 2.99 times more likely to have individuals with no mf compared to the zero IVM intake group (OR = 2.99; 95 % IC [2.19–4.08], P < 0.0001; Table 5). The opposite was observed with infected individuals (Fig. 5). The proportion of individuals with mf load ranging from 1–50 mf/ss decreased from 50.9 % in the untreated group to 31.7 % in the ≥7 treatment round groups (OR = 0.45; 95 % IC [0.33–0.61], P < 0.0001; Table 5). The same trend was observed with [51–100 mf] and [>100 mf] groups (OR = 0.15; 95 % IC [0.05–0.45], P = 0.001; and OR = 0.22; 95 % IC [0.06–0.82], P = 0.025, respectively; Table 5; Fig. 5). This relationship was also found with the entire study population (Table 6; Fig. 6) but not with children (Fig. 4).

**Table 5:** Odds of having adults with defined mf loads in the different IVM intake groups (Adults only)

Odds	IVM intake (Rounds)	Number examined	Number confirmed^a^	OR (95 % confidence interval)	*P* value
Odds of having people with 0 mf load	[0]	285	114	Reference	
	[1–3]	640	296	1.29 [0.97–1.71]	*P* = 0.078
	[4–6]	543	325	2.23 [1.67–2.99]	*P* < 0.0001
	≥7	419	279	2.99 [2.19–4.08]	P < 0.0001
Odds of having people with 1–51 mf load	[0]	285	145	Reference	
	[1–3]	640	312	0.91 [0.69–1.21]	*P* = 0.55
	[4–6]	543	208	0.59 [0.45–0.80]	*P* = 0.001
	≥7	419	133	0.45 [0.33–0.61]	*P* < 0.0001
Odds of having people with 51-100 mf load	[0]	285	17	Reference	
	[1–3]	640	12	0.3 [0.14–0.]64	*P* < 0.002
	[4–6]	543	5	0.14 [0.05–0.4]	*P* < 0.0001
	≥7	419	4	0.15 [0.03–0.4]	*P* = 0.001
Odds of having people with >100 mf load	[0]	285	9	Reference	
	[1–3]	640	17	0.99 [0.45–2.2]	*P* = 0.98
	[4–6]	543	5	0.28 [0.09–0.86]	*P* = 0.02
	≥7	419	3	0.22 [0.06–0.82]	*P* = 0.025

^a^Number of individuals identified in each mf load group with respect to IVM intake

**Table 6:** Odds of having people with defined mf loads in the different IVM intake groups (Overall study population)

Odds	IVM intake (Rounds)	Number examined	Number confirmed^a^	OR (95 % confidence interval)	*P* value
Odds of having people with 0 mf load	[0]	367	151	Reference	
	[1–3]	956	464	1.35 [1.05–1.72]	*P* = 0.016
	[4–6]	615	357	1.98 [1.52–2.57]	*P* < 0.001
	≥7	426	282	2.85 [2.09–3.70]	*P* < 0.001
Odds of having people with 1-51 mf load	[0]	367	178	Reference	
	[1–3]	956	451	0.94 [0.75–1.21]	*P* = 0.66
	[4–6]	615	244	0.70 [0.54–0.90]	*P* = 0.007
	≥7	426	137	0.50 [0.37–0.67]	*P* < 0.001
Odds of having people with 51-100 mf load	[0]	367	24	Reference	
	[1–3]	956	16	0.24 [0.13–0.47]	*P* < 0.001
	[4–6]	615	7	0.16 [0.70–0.38]	*P* < 0.001
	≥7	426	4	0.35 [0.04–0.39]	*P* < 0.001
Odds of having people with >100 mf load	[0]	367	14	Reference	
	[1–3]	956	25	0.67 [0.34–1.32]	*P* = 0.251
	[4–6]	615	7	0.29 [0.11–0.72]	*P* = 0.008
	≥7	426	3	0.18 [0.05–0.63]	*P* = 0.007

^a^Number of individuals identified in each mf load group with respect to IVM intake

**Fig. 7:**
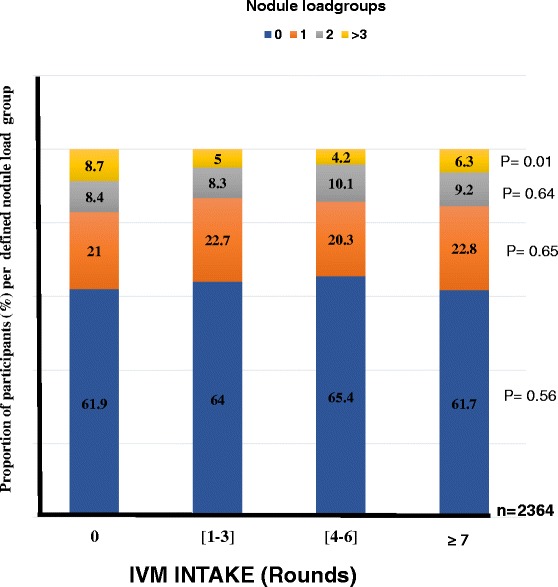
Changes in the proportion of study population in defined nodule load groups in relation to IVM intake profile. (Number of people examined per IVM intake groups: [0 time] = 367; [1-3 times] = 956; [4-6 times] =615; [≥7 times] =426). P-value given for each defined nodule load group Significance level set at 5 %

No clear relationship between treatment and nodule intensity was found. Of 1,504 individuals with zero nodules, 61.9 % had never taken ivermectin. This proportion was similar for all the other IVM intake groups (P = 0.168). The same observation was made with people with nodules. For individuals with > 7 times IVM intake, 6.3 % had > 3 nodules, this proportion was not significantly different from 8.7 % that of people who had never taken IVM (P = 0.254, Fig. 7).

## Discussion

This is the first study designed to assess the use of community members’ oral declaration on ivermectin intake to determine the impact of treatment on parasitological indicators of onchocerciasis in the rain forest area of Africa where *Loa loa* co-exists. This study has shown adherence to treatment to be inadequate in the study area; the prevalence of the disease based on the mf presence remained high (47.0 %) after more than a decade of mass treatment with ivermectin. It has also shown a negative association between ivermectin intake and disease prevalence as well as intensity. Previous studies have demonstrated long term treatment with ivermectin to be associated with remarkable reduction in disease prevalence and intensity [17–19], however none of these studies analysed the data in relation to individuals’ IVM intake. Individuals who declared not to have taken Ivermectin, or those who declared to have taken it just few times, were those with higher prevalence and intensity of infection. Therefore, the parasitological data validated the information from interview. This was an indication of the reliability of participants’ oral declarations. Others studies have used this approach to assess the barriers to coverage and compliance to MDA for lymphatic filariasis [14, 27] and the effect of adherence on the impact of MDA for elimination of lymphatic filariasis [13]. Treatment registers are not always available for information to be retrieved from [27]. In our setting, registers are kept at two different levels (health system and community). Normally, each year just before mass drug distribution, the health system gives registers out to community drug distributors (CDDs). CDDs share the drugs, fill the registers and send them back to the health system. However, this is not always the case. More often than not, some CDDs fail to submit completed registers to the health system and this causes a major problem of register availability. Registers kept at the community level sometimes are not properly handled and this constitutes another challenge, i.e. that of the quality of retrieved registers. Some registers are returned with missing pages or pages not properly filled, making the exploitation of the data difficult and unreliable [27]. This study has demonstrated that when unavailable (or unreliable), registers could be bypassed by relying on community members’ oral declaration of IVM intake as a proxy to assess adherence to treatment.

In the present investigation, there was a lukewarm attitude towards ivermectin in the study area. Of the examined participants, up to 15.5 % had never taken the drug. The majority had taken the drug just 1–3 times out of the 10 and 12 rounds of ivermectin mass distribution. Moreover, only 18 % of the study population had received IVM 7 times or more. This proportion was very small in children (1.5 %) and could be related to the fact that they had not had exposure to the same number of MDA as adults. These low adherences in meso- and hyperendemic areas (as in the present setting) may constitute a potential reservoir of onchocerciasis infection that could hinder efforts to control the disease [13]. It remains unclear whether ivermectin treatment can be halted if the parasite reservoir has not been completely eradicated [33]. This is a, if not the critical issue to resolve since MDA against onchocerciasis requires at least 15 years to achieve elimination [12, 34].

The low adherence observed in this study could be attributed to fear of side effects as reported by many authors [13, 14, 26]. Besides itching, it has been shown that people co-infected with *Loa loa* and having high *L. loa* mf load develop encephalopathy following ivermectin treatment [14, 22–24]. Such manifestations were reported at the onset of mass ivermectin treatment in our setting [28]. People remain unwilling to accept IVM treatment due to the risk of side effects [26, 35].

Males stuck to the treatment regime relatively more than females and adults more than children. These findings corroborate those of Brieger et al. [25] who reported age and sex as factors explaining variations in adherence to IVM treatment. Communities with low adherence indicate a low level participation of the communities in CDTI and possibly difficulties in eradication efforts whereas a high adherence would indicate high level of community participation in drug distribution and administration leading to possible decrease in microfilaria prevalence rates and loads, ultimately meeting APOC objectives.

The prevalence of onchocerciasis in our study area was still high 47.0 % for mf and 36.4 % for nodules despite a decade of ivermectin treatment. This can be justified by the low adherence found in the study areas. In addition to high pre-control endemicity levels and high vectorial capacity reported in the area [36–38], the perennial presence of ecological factors, such as fast flowing rivers that strongly favour the breeding and development of the black fly, help maintain continuous transmission and hence high prevalence of infection [21].

Slightly more males were infected than females. This is in line with findings of Moyou et al. [39] and could be linked to their daily activities, i.e. cocoa and palm farming, which are the main occupation of males and source of income in our study area. Another reason could be the fact that men prefer working with exposed bodies and are thus prone to more bites than (covered) females.

Hormonal differences between the sexes have been suggested to account for the relatively higher resistance of females relative to males under similar ecological conditions [39, 40]. The mf prevalence and WMMfD in children were relatively higher than in adults. Children’s life style involves playing around the rivers, bushes and, above all, they have no sense of protection of the body with clothing from exposure to fly bites. Duke & Moore [41] reported these findings many years ago and it seems to still be the case particularly with children living in remote areas.

Ivermectin treatment has reduced the endemicity of onchocerciasis in the study area from a pre-treatment mf prevalence and intensity of 92.7 % and 53.6 mf/ss [39] to 47 % and 4.82 mf/ss respectively. These findings show that if adherence to ivermectin treatment is maintained in communities, parasitological indicators of onchocerciasis would drastically reduce. In this study, individuals who had never taken the drug were found to have a significantly higher mf prevalence (58.9 % overall and 60 % for adults) compared to (33.8 % overall and 33.3 % for adults) for those who had taken the drug ≥ 7 times. The same trend was found with the WMMfD. There was a significant negative association between the above-mentioned indicators and IVM intake. Our results clearly show the important influence of IVM treatment adherence on residual infection rates after MDA. It was not surprising that residual infection rates were highest in people who reported that they had never taken MDA. Although 1–3 rounds of ivermectin can clear the skin of microfilariae, 7 or more rounds of MDA had more significant effect on mfs. Similar results have been reported from a study on the effect of adherence on the Impact of MDA for Elimination of Lymphatic Filariasis in Egypt [13].

These observations highlight the importance of adherence for MDA and the following could be deduced:

(i) The elimination of the disease in an area will depend to a great extent to the proportion of high adherents relative to that of low adherents as they represent a disease reservoir for the community [14, 25]. Moreover, children who have never taken IVM were found to have the highest intensity of infection among the study population and as such constituted a danger for the community. This risk is further heightened because those under 5 years are excluded from MDA programmes. Their life style has previously been described as a factor favouring transmission [41].

(ii) Oral declaration on IVM intake could be used as proxy to assess the impact of treatment at individual level when registers are lacking. Our results revealed that in cohorts that have received ivermectin 7 times or more, there is a higher likelihood that more than 60 % will be without mf in the skin compared to 44 % in the pool with zero IVM intake (Fig. 4). This implies that knowledge of treatment adherence in a community would give a rough estimate of the endemicity of the disease in that community, or area.

If such a study is carried out in different rain forest areas, a mathematical model could be developed for the prediction of the proportion of healthy (with no microfilaria) versus infected individuals in the community based on IVM intake. This would be a very helpful and non-invasive approach to rapidly delineate areas in great needs of alternative strategies for the elimination of onchocerciasis in case that a macrofilaricide was adopted for mass treatment for onchocerciasis. Rapid assessment procedures have been useful for the identification of priority communities for the Ivermectin MDA (REA/REMO) or communities or regions at risk for ivermectin MDA because of the presence of *Loa loa*. [6, 7, 10, 30, 42–45].

Although there was a significant reduction in the proportion of infected people with IVM intake, people with high mf load (>50 mf/ss) were found in all IVM intake groups. This persistent high mf load, even in individuals with more than 7 rounds of treatment, could be linked to the way the drug was taken. Those people may have been taken the drug in a discontinuous manner and by so doing facilitate mf load to build up before a second treatment is taken. Another reason could be a high pre-treatment endemicity level in addition to the presence of reservoir of infection (non-adherents and children under 5 years). Pre-treatment mf prevalence and intensity were demonstrated to be as high as 92.7 % and 53.6 mf/ss in our setting respectively [39].

In addition to adherence rates, the ONCHOSIM prediction model for onchocerciasis elimination takes into account variability in vectorial capacity and pre-control endemicity. It stipulates that the higher the pre-control endemicity level, the longer the time required to achieve disease elimination [12, 34]. This might be the case in the area surveyed [21].

Many authors have suggested a twice per year treatment strategy accompanied by an increase in therapeutic coverage to at least 85 % in areas with continuous transmission after several years of control efforts using Ivermectin [21, 46]. This strategy can lead up to four rounds of treatment yearly and has contributed tremendously to the elimination of onchocerciasis transmission in some settings [47–49]. This approach does not only reduce transmission but also reduces the number of years of treatment needed to achieve elimination of onchocerciasis [20, 34]. In an area where the perceived fear of SAEs is well rooted in the population, the biannual treatment with ivermectin may not be of significant impact. This might be the case in the south west region of Cameroon, where the systematic non-adherence to ivermectin coupled to low adherence sum up to more than 60 % of the population. In such situation, alternative treatment may be required as ivermectin alone may never lead to the elimination of onchocerciasis. Several alternative strategies can be envisaged in this situation. The first one could be the test and treat strategy in which, the population is screened for onchocerciasis and those found positive for *O. volvulus* microfilariae are treated with a macrofilaricide drug. The choice of such macrofilaricide drug deserves an important consideration. It should kill the adult *O. volvulus*, without killing drastically the *L. loa* microfilariae. Drugs that deplete *Wolbachia* spp., the endosymbionts found in several filarial species, have those properties [50–52]. The non-effect on *L. loa* microfilariae are justified by the absence of *Wolbachia* spp. from *L. loa* [53]. Antibiotics of tetracycline family have been demonstrated in several studies to efficiently kill adult *O. volvulus* [50, 51, 54–56] with no effect on *L. loa* microfilariae [51, 53]. Indeed, Doxycycline, has been successfully administered at large scale level for the treatment of onchocerciasis in area of co-endemicity of *L. loa* in Cameroon in a pilot study, with a high treatment adherence from the population, significant impact on the parasitological indicators of onchocerciasis and lack of severe adverse events [57, 58]. The second alternative strategy option that could be envisaged in this rain forest area of south west Cameroon is the vector control, targeting larvae of *Simulium* spp. in the breeding sites. The rain forest area of South west Cameroon is characterised by numerous rivers, getting their sources from the flank of mountains and flowing fast down the hills, constituting excellent breeding places for *S. squamosum* and *S. yahense* [21]. More important is the fact that the level of water from those rivers is maintained sufficiently high during dry season to allow continuous breeding throughout the year. This is an important consideration as the epidemiological implications of the continuous breeding of vectors is the high annual transmission potential which ensures that even residual microfilariae of *O. volvulus* in the skin can be picked up by *Simulium* spp. that are constantly present. Vector control using ground applications of Temephos, an insecticide with low environmental hazards risk, has been used successfully in the west Africa by the Onchocerciasis control programme, in the Equatorial Guinea (Bioko Island) and Uganda by the African programme for Onchocerciasis control [59, 60].

The impact of IVM intake was most readily reflected in mf prevalence and intensity rather than nodule prevalence. No association between IVM intake and nodule burden was found. This could be attributed to the fact that ivermectin is a microfilaricidal drug and as such has minor effects on adult worms in nodules hence the little variations in nodule prevalence and intensity with treatment [21, 61, 62]. In the savannah areas, there are data both published and unpublished showing that nodule rate reduces with the IVM MDA [63]. The persistent nodule rate in this area, could also be explained by continuous transmission.

Another important point to stress on, is the fact that no association between adherence to treatment and mf prevalence and intensity was found in children. This could imply that children are not as good as adults at recalling the number of times they have taken the drug. Also, they could have given wrong responses thinking they would be blamed if they declared they had not been taken the drug hence, the necessity of sensitising adolescents (11 years and above) prior to using the approach described in this paper. Adherence data for children could also be obtained from parents or guardians of enrolled children [13]. For the present study, only children less or equal to 10 years of age were assisted by their parents or guardians assuming older individuals would be able to give reliable responses.

Additional studies are required to understand the reasons for non-adherence to MDA, to refine health education message(s) to target the non-adherents, and to put in place alternative approaches for *crossing the finish line*. These might include vector control, mass treatment with macrofilaricides if available or test and treat activities that permit selective treatment of people with residual infections. Data from the present study have shown that knowledge of the adherence to MDA in a region could give a broad idea about the onchocerciasis endemicity level in the area. Identifying communities of greatest needs for control would constitute a prerequisite for implementing the above-mentioned alternative approaches for crossing the finish line. The approach described herein could be a convenient non-invasive way of achieving that task. This approach could also be used to rapidly assess CDTI projects that had issues (SAEs) at the onset or during the implementation of the control programme.

## Conclusion

This study was designed to assess the relationship or association between oral declaration on the adherence to ivermectin treatment and the parasitological indicators of the infection. It has revealed that adherence to ivermectin treatment is not adequate in this rain forest area where *L. loa* also co-exists with *O. volvulus*. The prevalence and intensity of onchocerciasis remain high in individuals who have never taken ivermectin after more than a decade of MDA. There was a significant negative association between adherence to treatment and mf prevalence and intensity. This was an indication that participants’ oral declaration could be relied upon for the evaluation of the impact of long term treatment of onchocerciasis with ivermectin. However, there may be need to refine this approach through validation in other communities or context. There is need to implement alternative strategies in settings like the one described in this paper.

## Abbreviations

APOC: African Programme for Onchocerciasis Control
CDTI: Community-directed treatment with ivermectin
CMFL: Community Mf Load
IVM: Ivermectin
MDA: Mass Drug Administration
MF: Microfilaria
OCP: Onchocerciasis Control Programme
ONCHOSIM: Onchocerciasis transmission elimination simulation model
RAPLOA: Rapid Assessment Procedure for Loiasis
REMO: Rapid Epidemiological Mapping of Onchocerciasis
SAEs: Severe adverse effects
WMMfD: Williams Mean Mf Density
